# Modulation and bioinformatics screening of hepatic mRNA-lncRNAs (HML) network associated with insulin resistance in prediabetic and exercised mice

**DOI:** 10.1186/s12986-021-00600-0

**Published:** 2021-07-20

**Authors:** Fatemeh Kazeminasab, Sayed Mohammad Marandi, Maryam Baharlooie, Mohammad Hossein Nasr-Esfahani, Kamran Ghaedi

**Affiliations:** 1 Department of Exercise Physiology, Faculty of Sport Sciences, University of Isfahan, Hezar Jerib Avenue, Azadi Sq., Isfahan, 81746-73441 Iran; 2 Department of Physical Education and Sport Sciences, Faculty of Human Sciences, University of Kashan, Ravand Street, Kashan, 87317-35153 Iran; 3 Department of Cell and Molecular Biology and Microbiology, Faculty of Biological Science and Technology, University of Isfahan, Hezar Jerib Avenue, Azadi Sq., Isfahan, 81746-73441 Iran; 4 Department of Animal Biotechnology, Cell Science Research Center, Royan Institute for Biotechnology, ACECR, Royan Street, Salman Ave, Khorasgan Square, Jey Ave, Isfahan, 81593-58686 Iran

**Keywords:** Endurance exercise, Insulin resistance, lncRNA, Non-alcoholic fatty liver disease, Prediabetes

## Abstract

**Background:**

Insulin resistance is associated with prediabetes and further progression to type 2 diabetes mellitus (T2DM). This study aims to investigate novel hepatic lncRNAs associated with key genes in insulin resistance in prediabetes.

**Methods:**

In the bioinformatics phase, we have collected screened a pool of lncRNAs and mRNAs according to their potential association to prediabetic condition. We performed pathway analysis of mRNAs, using DAVID tool based on KEGG repository data. Then, we used Python programming language to get a subset of lncRNAs located in 50 kb proximity with high-fat (HF)-responsive mRNAs. In the experimental phase, prediabetic mice model was established by the treatment of HF diets for 12 weeks. After this treatment, HF-fed animals were divided into two groups of endurance exercised or sedentary, both continuing on the HF diet for 8 weeks. Besides, a group of diabetic mice was treated using a HF diet for 8 weeks followed by injection with STZ solution and then a HF diet for another 4 weeks.

**Results:**

We found three genes having paired lncRNAs annotated in insulin resistance pathway. Their hepatic expression levels were altered in prediabetic condition as upregulation of *Srebf1* was associated with *GM38501*, upregulation of *Pck1* was associated with *Ctcflos* and GM36691, downregulation of *Cpt1b* was associated with *GM44502*. All of these expression patterns were replicated in diabetic mice, correlated positively with their predicted lncRNAs. Interestingly, exercise reversed their expression patterns.

**Conclusions:**

We suggest that the expression pattern of the hepatic mRNA-lncRNA (HML) network in prediabetic state undergoes similar modification to that of diabetes.

**Supplementary Information:**

The online version contains supplementary material available at 10.1186/s12986-021-00600-0.

## Introduction

Prediabetes, defined as having elevated fasting glucose and impaired glucose tolerance, is rising in prevalence even faster than “Type 2 diabetes mellitus” (T2DM). It usually does not cause symptoms but people with prediabetes often have obesity (especially abdominal or visceral obesity), dyslipidemia with high triglycerides and/or low HDL cholesterol, and hypertension [1]. Prediabetic model mice can be diagnosed by measuring Glycated hemoglobin A1C (HbA1c), fasting glucose, and glucose tolerance test (GTT) [2]. In a person with prediabetes, the level of fasting blood glucose is 110–125 mg/dL (6.1–6.9 mmol/L), blood sugar level of 140–199 mg/dL 2 h after ingesting glucose solution, and HbA1c between 5.7 and 6.4%, i.e. 38.9 and 46.4 mmol/mol [3]. Unfortunately, in most cases prediabetes is ignored by the people and gradually it might irreversibly lead to T2DM [1, 4]. In T2DM a series of metabolic dysfunctions occur such as diminished insulin action and impaired insulin secretion, elevated hepatic glucose production, and increased insulin resistance (IR) [4]. Predominantly due to the obesity, IR is a main determinative factor associated with metabolic syndrome that leads to T2DM. In the IR condition, cells cannot respond adequately to the insulin, in almost all tissues [5]. Two key factors have been proposed to associate with the mechanism of insulin resistance; including obesity and fatty liver disease [6]. Non-alcoholic fatty liver disease (NAFLD) is one of the most common chronic liver disorders worldwide. Hepatic steatosis is a result of lipid accumulation in hepatocytes, which is linked to obesity, insulin resistance, and T2DM. It includes liver tissue alterations ranging from simple steatosis to non-alcoholic steatohepatitis (NASH), liver cirrhosis, and hepatocellular carcinoma [7]. Hierarchy to insulin resistance, an extreme accumulation of triglyceride (TG) in the liver occurs. This increase in TG plays an important role in the development of hepatic and systemic insulin resistance. Some researchers hypothesize that liver fat accumulation is a consequence, rather than a cause of peripheral insulin resistance in obesity. In skeletal muscle, peripheral IR will mainly affect a large portion of the total glucose uptake (> 80–90%), while in adipose tissue, IR will induce an impaired anti-lipolytic action of insulin and increased release of non-esterified fatty acids (NEFA) [8]. Numerous studies have shown that eating a high-fat diet leads to accumulation of hepatic fat, hepatic IR, development of peripheral IR, hyperinsulinemia, and impaired glucose tolerance [8–10].

Several animal studies have explored the beneficial effects of exercise on diabetes and prediabetes-associated pathophysiology. Studies revealed that forced exercise could decrease inflammation in the adipose and intrahepatic lipid deposits in the liver tissue [11–13]. Interestingly, in obese mice model of HF diet-induced IR that underwent an exercise program, IR reduced [14]. There is a need for an in-depth understanding of the molecular mechanism responsive for physical activity to confer its protective effect against IR. Such study helps development of novel therapeutic targets for T2DM, as well as understanding the potential mechanisms involved in the progression of prediabetes to T2DM, and ways to prevent this progression.

LncRNAs are an emerging class of non-coding RNAs with potential regulatory effects on target genes expression, particularly through transcriptional and post-transcriptional regulation of gene expression. Gene expression is regulated by lncRNAs at multiple levels. By interacting with DNA, RNA and proteins, lncRNAs can modulate chromatin structure and function and the transcription of neighboring and distant genes, and effect RNA splicing, stability and translation. Cis-acting lncRNAs, which constitute a substantial fraction of lncRNAs with an attributed function, regulate gene expression in a manner dependent on the location of their own sites of transcription, at varying distances from their targets in the linear genome. Through various mechanisms, cis-acting lncRNAs have been demonstrated to activate, repress or otherwise modulate the expression of the target genes [15]. The lncRNAs has been associated with a variety of metabolic conditions such as obesity [16–18] Type 1 diabetes [19] and T2DM [20–22] Nevertheless, association of lncRNAs to prediabetes condition is not well understood. Therefore, this study aims to investigate novel hepatic lncRNAs associated with key genes related to insulin resistance in prediabetes in the liver.

## Material and methods

### Bioinformatics passage

Through a vast bioinformatics survey, we collected liver metabolic-sensitive lncRNAs and gene expression datasets available in Gene Expression Omnibus (GEO), Mouse Genome Database (MGD), and literature exploring for prediabetes associated genes and lncRNAs. Next, we screened lncRNAs and mRNAs from datasets (GSE85439 and GSE94790), with differential expression in the liver of mice treated with high-fat diet, based on log FC > 2 compared to LF diet groups. Then, we examined the coding potential of lncRNAs resulted from analyzed GEO data, using the Coding Potential Calculator (CPC) database, and selected transcripts lacking strong coding potentials. We performed pathway enrichment analysis of total mRNAs using Database for Annotation, Visualization and Integrated Discovery (DAVID) *v*6.8 (https://david.ncifcrf.gov/) based on Kyoto Encyclopedia of Genes and Genomes (KEGG) repository data to screen the most potential pathways in HF-fed mice liver.

We obtained the chromosomal location of selected both lncRNAs and mRNAs sets from Ensembl genome browser Mouse GRCm38.p6, and assigned them to a Python programming language code [23, 24] to get a subset of lncRNAs and mRNAs located in 50 kb proximity. Eventually, we chose mRNAs of genes enriched in pathways associated with the metabolism of carbohydrates and lipids for further analysis.

Then, we constructed and visualized a network of predicted association of lncRNAs and mRNAs, even though analyzing this network for protein–protein interactions in Cytoscape software, using interaction scores from STRING DB *v*11.0. We analyzed the interaction network of genes enriched in the insulin resistance pathway, which were involved in more than two pathways. Finally, we chose hepatic mRNA-lncRNA (HML) pairs, which had the highest degree centrality score and were functionally annotated in a common cluster of carbohydrates and lipids metabolism as well as annotation in NCBI database.

### Animals

All experiments with animals were carried out according to the policy of the Ethics Committee of the University of Isfahan and Royan Institute, which was compatible with Canadian Council on Animal Care guidelines (Ethics code IR.ACECR.ROYAN.REC.1398.45).

After a one-week acclimatization, six-week-old male C57BL/6 J mice (n = 30) were kept under controlling temperature (23 ± 1 °C), moisture (50 ± 3%), and a photocycle of the 12 h light-12 h dark cycle. Diet and water were provided ad libitum. Glucose tolerance tests (GTT) were conducted by fasting the mice for 4–5 h, followed by intraperitoneal delivery of 2 g/kg D-glucose in a 30% Phosphate-buffered saline (PBS) solution. Blood glucose was tested after 0, 30, 60, 90, and 120 min [25]. For insulin tolerance test (ITT), mice were fasted for 4–5 h and blood sample was taken, followed by intraperitoneal injection of insulin (0.75 IU/kg). Blood sampling was done at 0, 30, 60, 90, and 120 min after insulin injection. The samples were analyzed for blood glucose, and the area under the curves (AUC) during the ITT was calculated.

### Prediabetic model induction

For three months, the mice were fed with the low fat (LF) diet or the high-fat (HF) diet (60% of the energy was supplied from fat). HF diet (20% kcal from carbohydrate, the 20% kcal from protein, 60% kcal from fat with 32% saturated, 35% monounsaturated and 33% polyunsaturated fatty acids); LF diet (70% kcal from carbohydrate and 20% kcal from protein, 10% kcals from fat with 23% saturated, 30% monounsaturated and 47% polyunsaturated fatty acids); high-fat diet. Prediabetes was induced in the animals fed by the HF diet. These mice were used as the diet-induced models of obesity and the associated metabolic disorders [26, 27]. After 12 weeks of intervention, to confirm the induction of prediabetes in a mouse model, GTT, and ITT were performed after 4–5 h of fasting.

### Diabetic model induction

The mice were first fed with a high-fat diet for 8 weeks, followed by intraperitoneal injection (i.p.) with STZ solution (140 mg/kg, dissolved in 0.1 M sodium citrate buffer) [28, 29]. After injection, the mice were fed by HF diet for extra 4 weeks. Blood glucose (BG) level was tested from the tip of the tail. The diabetic mice model establishment was considered successful when BG levels were higher than 11.2 mmol/L. During this procedure, the control group (Ctrl) was fed with a LF diet and was injected with 0.1 M sodium citrate buffer (without STZ injection). Following the aforementioned 12-week intervention, GTT and ITT were performed after 4–5 h of fasting.

### Exercise training simultaneous with HF diet

After 12 weeks, the HF-fed mice (prediabetic group) were divided into two groups: 1. HF diet-Exercised (HF-Exe), 2. HF diet-Sedentary (HF-Sed) (Fig. 3A). One training group exercise was carried out using a treadmill for 8 weeks. The exercise protocol for two months was as follows: In the week 13 of the study, an adaptation protocol was carried out for the exercise groups, such that each mouse was placed on the treadmill starting with 17 m/min for 15 min on the first day. Then, the exercise duration was increased daily to the final 45 min (17 m per min for 45 min at the end of the thirteenth week). In the weeks 14–21, the exercise speed was progressively increased by 2 m/min every two weeks to the final threshold of 23 m/min. The duration of exercise in this period was 45 min per section, 5 days per week, one session per day. During this study, the inclination angle was 0% gradient. This situation indicated the moderate-intensity resembling about 70% vO_2_max [26, 30]. While the LF diet-fed mice (control group) were fed the long-term LF diet and were inactive. After 8 weeks of intervention, to confirm the prediabetes condition in HF-Sed, fasting blood sugar, and HbA1c were performed after 12 h fasting. The day after measuring the tests, prediabetic mice were sacrificed.

### Dissected liver samples and histological analysis

Following the aforementioned 8-week intervention, the body weights of the mice were measured on the day of sacrifice. Twelve hours after fasting, the animals were sacrificed, and the liver tissue was dissected.

For histological evaluation, representative hepatic sections were cut, fixed in 4% paraformaldehyde-PBS, inserted in paraffin, and stainted with hematoxylin and eosin (H&E) following standard proceeding. Semiquantitative evaluation of steatosis in liver tissue from five mice per group was done blinded by an experienced histopathologist according to the non- steatosis score defined by Brunt et al. (1999) [31]. Representative pictures were taken using an Olympus BX45 microscope (Olympus Deutschland, Germany) with a camera (Micro Optimal, Germany). Liver samples were graded according to Brunt et al. (1999) as follows: 0–3 based on percent of hepatocytes in the biopsy involved (0: no steatosis; 1: up to 33%; 2: 33–66%; 3: > 66%) zonal distribution of steatosis was noted [31].

### HbA1c, Glucose, insulin, triglyceride, and HOMA‐IR

Mouse immunoreactive HbA1c was determined using DCA 2000 analyzer (Bayer, Elkhart, IN). This system automatically measures both HbA1c and total hemoglobin in an approximately 5-μL blood sample [32, 33]. Approximately 10–15 μL of blood was collected via a vein of mouse and measured HbA1c.

Blood collection was carried out after the 12 h in fasting state, on the final day of the second intervention (Exercise). To measure fasting blood glucose, samples were assessed via glucose diagnostic reagents (Sigma, St. Louis, MAK013). To evaluate insulin levels, blood was centrifuged for 15 min at 3 × 10^3^*g*, and plasma was removed. The insulin levels were measured by the ELISA kit (mouse insulin Elisa, Alpco, 80-INSMS-E01). The homeostatic model assessment of insulin resistance **(**HOMA‐IR) as a scale of insulin resistance was determined by using the fasting blood glucose and insulin levels. The following equation was used to compute the HOMA‐IR value: Blood (plasma Insulin (μU/mL) × fasting Glucose (mg/dL) /405 [34].

To determine the concentration of triglyceride (TG), enzymatic methods were used in a calibrated biochemical analyzer (Hitachi 902 Automatic analyzer, Roche Diagnostics, USA) as follows. TG was measured by assessment of the produced H_2_O_2_ [35].

### RT-qPCR analyses for mRNA level assessment

After RNA extraction, cDNA was synthesized using TAKARA cDNA synthesis kit according to the manufacturer protocol and was stored at − 70 °C for further qRT PCR tests [26]. We used SYBR green method for qRT PCR using ABI (step one plus) equipment. Primers for mRNAs and lncRNAs were designed using Beacon designer, Oligo7, and NCBI blast tools. Efficiency and specificity of primers were confirmed through gradient PCR followed by gel electrophoresis. The primer pairs are presented in Additional file 4: Table 1. Analysis of relative expression levels was calculated by ΔΔCT method and data was normalized to *18SrRNA* expression levels.

### Statistical analysis

All data are shown as means ± SEM. The analysis of variance (ANOVA) was conducted to determine the influence of the aerobic exercise and diet for all parameters. Also, the results were evaluated statistically by Pearson correlation to find the relation between genes and lncRNAs expression. In the experiments of the present research, *p* value < 0.05 was considered as statistically significant.

## Results

### Bioinformatics results

Analysis of the GEO datasets (GSE85439, GSE94790), in addition to the results of MGD dataset mining, and literature survey suggested several coding genes and lncRNA sets were associated to prediabetes, revealed a total of 400 differentially expressed genes (Gene set 1) and 236 lncRNAs in the liver of mice treated with long term high-fat diet (Additional file 1: Fig. 1).

**Fig. 1:**
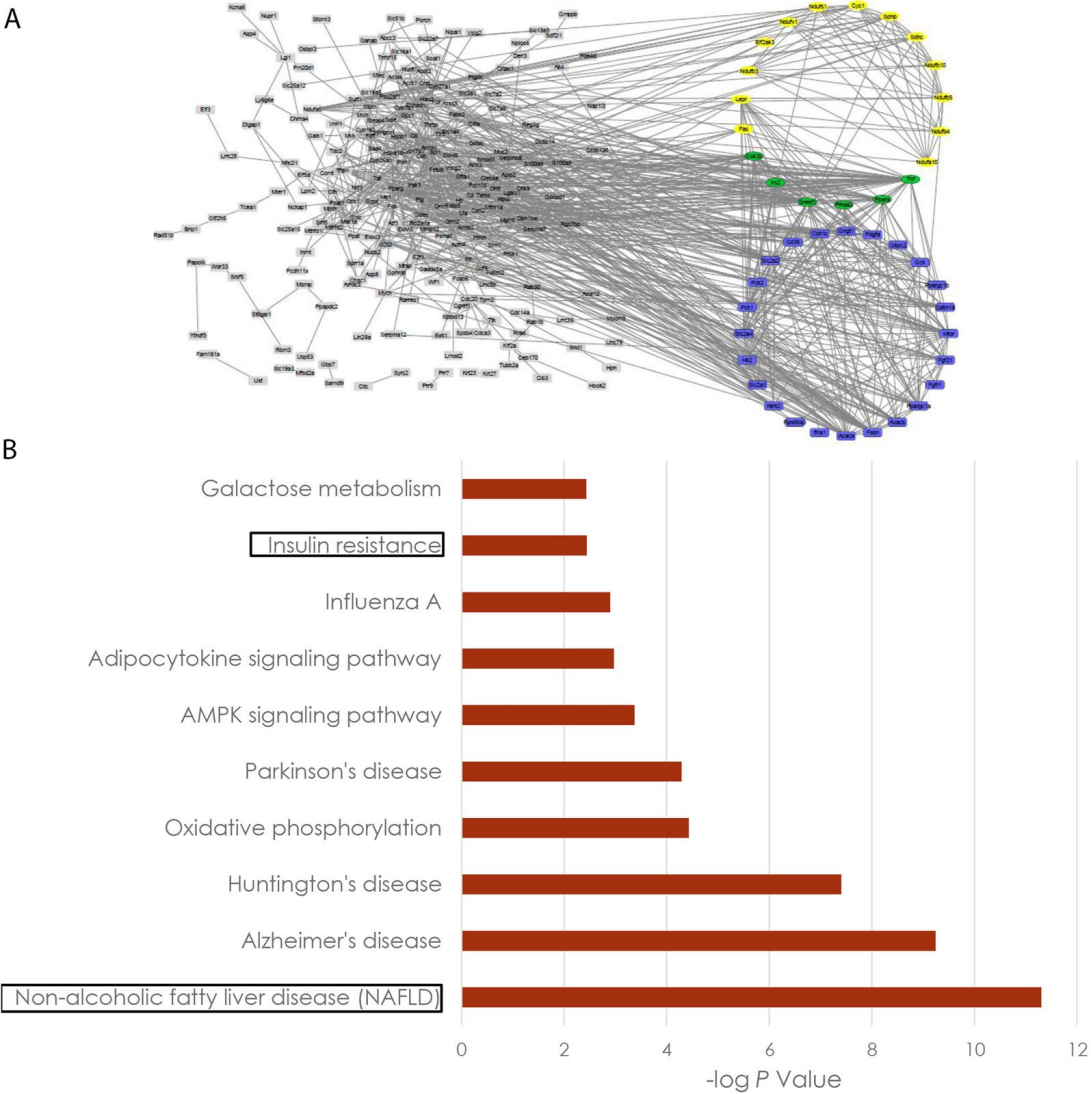
Pathway enrichment and interaction network of prediabetes associated genes. **A** Total prediabetes associated gene list gathered from literature, MGD, and GEO analysis enriched with DAVID online tool, and the PPI network drew from interaction scores. **B** NAFLD and insulin resistance as two of the top ones related to prediabetes condition. (DAVID: The Database for Annotation, Visualization and Integrated Discovery, PPI: Protein–protein interaction, NAFLD: Non-alcoholic fatty liver disease). The upper circle represents genes in NAFLD, while the lower one depicts insulin resistance associated with enriched genes. Yellow, blue, and green colors show lipid metabolism, carbohydrate metabolism, and both lipid and carbohydrate metabolism associated with genes, respectively

In the next step, the functional clustering analysis showed the enrichment of gene set 1 in pathways related to the pathogenesis and complications of prediabetes, including NAFLD, carbohydrate, and lipid associated metabolic pathways (Fig. 1).

**Fig. 2:**
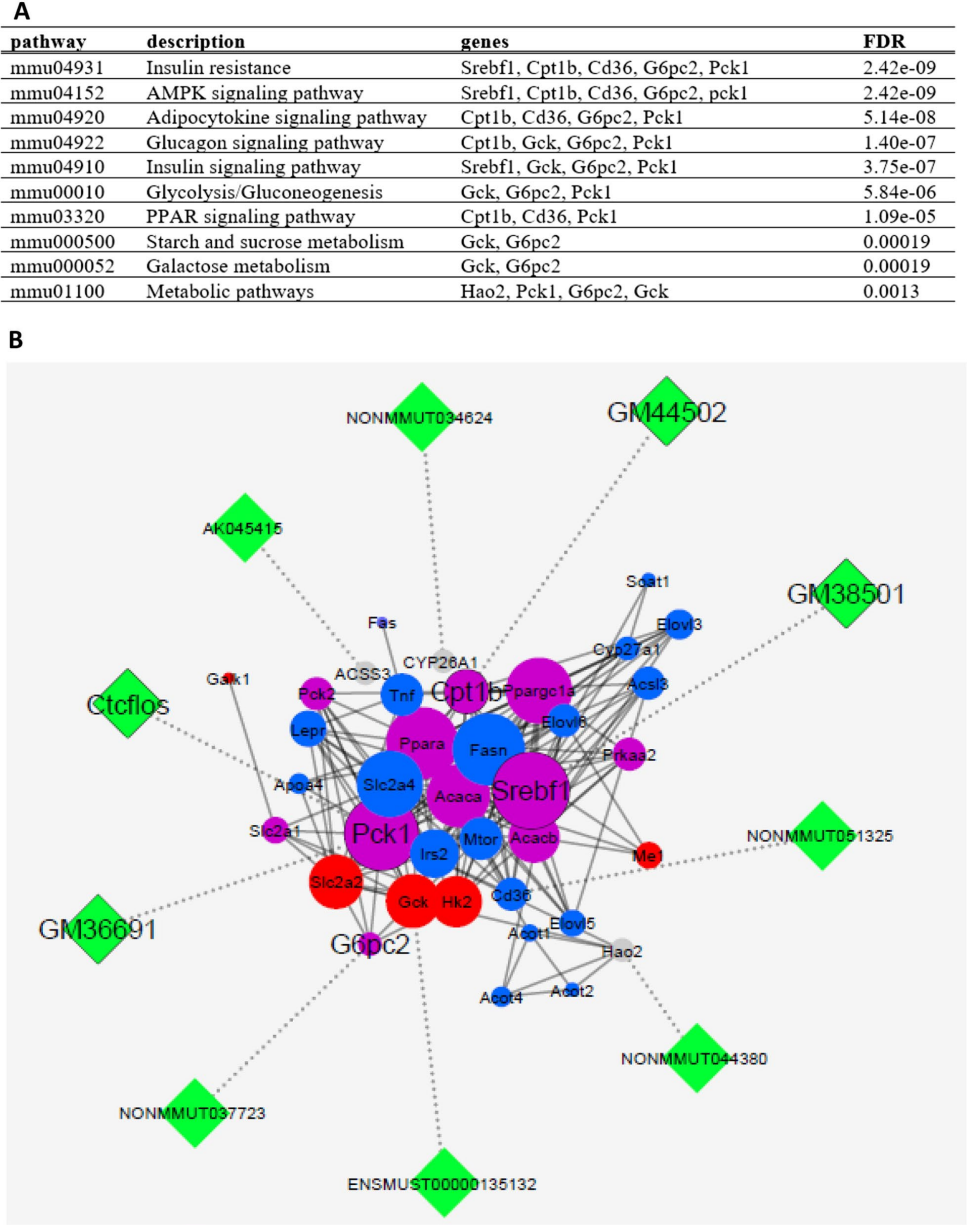
Interaction network of genes and predicted lncRNAs (HML). **A** We shortlisted genes according to three criteria: having the highest degree centrality values, belonging to both categories of lipid or carbohydrate metabolism, and having paired lncRNA in their proximity. **B** Python programming revealed 10 mRNA-lncRNA pairs within 50 kb proximity

Pathway enrichment of gene set 2 revealed insulin resistance as the most strongly enriched pathway followed by energy homeostasis related pathways (Fig. 2A, see also Additional file 2: Fig. 2). The gene set 2 refers to genes resulted from python programming on gene set 1, which were assigned to enrichment analysis in pathways associated with the metabolism of carbohydrates and lipids. Python programming with an input of chromosomal location of selected genes resulted in gene set 2 within 50 kb proximity to HF-associated lncRNAs. Then Gene set 2 were enriched in pathways associated with the metabolism of carbohydrates and lipids. Further analysis on the constructed network and functional clustering showed 10 hub mRNA-lncRNA pairs (Fig. 2B). Among them, five genes having paired lncRNAs were annotated in insulin resistance pathway and 4 of them were common in carbohydrate and lipid metabolism pathways. Finally, three of these genes had lncRNAs annotated in the NCBI genome browser (*Srebf1* was predicted to be associated with *GM38501*, *Cpt1b* associated with *GM44502*, and *Pck1* associated with *Ctcflos*, and *GM36691*). These three genes were key players in lipogenesis, lipolysis, and gluconeogenesis pathways, respectively. These pathways have been reported to be altered in NAFLD and IR [36–38]. We aimed to focus on pathways which were relevant on the liver tissue. Therefore, we chose NAFLD and IR among the best scored pathways (the least –log *p* values). As the aim of our study was to investigate hepatic mRNAs-lncRNAs (HML) network in prediabetes conditions. These two pathways NAFLD and IR were shown to play an important role in prediabetes and T2DM. Of note, we found adipocytokine signaling pathway in our bioinformatics enrichment analysis, which is not specific to the liver tissue, but could explain some of the events upon exercise on prediabetes and NAFLD.

### Experimental results

#### The effect of the high-fat on body mass, blood glucose, insulin in prediabetic mice

**Fig. 3:**
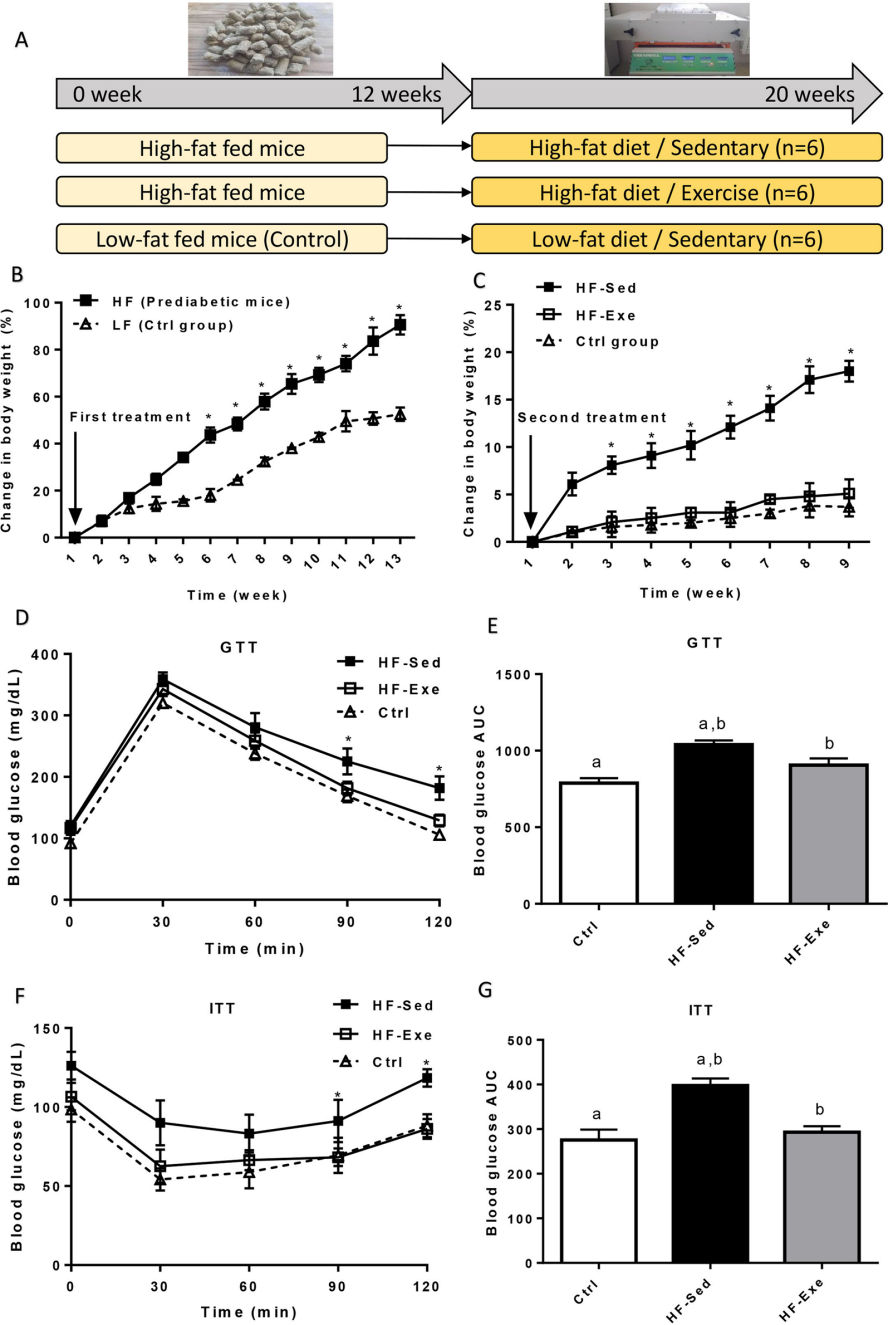
Body weight, blood glucose, and insulin monitored throughout the treatment of HF diet and exercise. **A** A schematic demonstration of the protocol with two interventions used for this study. **B** Changes in body weight in mice following the execution HF and LF diet in the first treatment. **C** Changes in body weight in all groups. **D** Glucose tolerance test (GTT) on control, HF-Sed, and HF-Exe mice after 20 weeks intervention. **E** The area under the curve (AUC) for the glucose tolerance test. **F** Insulin tolerance test (ITT) on control, HF-Sed, and HF-Exe mice after 20 weeks intervention. **G** The area under the curve (AUC) for the insulin tolerance test. *p < 0.05. All data are shown as means ± SEM, n = 6 per group

Figure 3A shows a protocol with two treatments which were applied in the present study. At the start point of the first treatment (3-month feeding HF and LF), no difference was detected in the body weight (B.W.) of animals. However, the B.W. had a significant increase in the mice fed by HF, as compared with the LF diet-fed counterparts (Fig. 3B). Figure 3C shows that prediabetic mice in the HF-Sed group had more body weight gain than the HF-Exe group (*p* < 0.05). Also, the body weight of the diabetic mice was reported in Additional file 3: Fig. 3A. The body mass of prediabetic and diabetic mice were almost the same.

In GTT, levels of the blood glucose increased significantly by administration of glucose. Subsequently, the blood glucose levels decreased gradually returning to the same levels at 120 min. The significant differences were observed in the HF fed mice at 90 and 120 min compared to the control group. This was corroborated by significantly increased glucose AUC values in HF fed mice (*p* < 0.05) as compared to control group (lean mice) (Fig. 3D, E). Also, AUC values were significantly decreased (*p* < 0.05) in HF-Exe vs. HF-Sed (Fig. 3D, E). In the ITT, HF fed mice (prediabetic mice) demonstrated higher blood glucose level in response to exogenous insulin load (*p* < 0.05) than control group (Fig. 3F, G).

#### The effect of the high-fat on triglyceride, HbA1c, and HOMA-IR in prediabetic mice

Triglyceride, fasting blood glucose, and HbA1c data indicate that the HF-Sed mice were prediabetes before being sacrificed. And the prediabetic conditions improved after exercise in the HF-Exe group compare to the HF-Sed mice (Table 1).

**Table 1:** The levels of fasting blood glucose, insulin, triglyceride, HbA1c and HOMA-IR before sacrifice mice

Variables	Ctrl	HF-Sed	HF-Exe
Fasting blood glucose (mg/dL)	94.1 ± 3.4	122 ± 6.5	96.3 ± 7.8
Fasting blood insulin (ng/mL)	1.2 ± 0.05	5.7 ± 1.4	1.8 ± 0.09
HbA1c (mmol/mol)	26.3 ± 1.1	40.1 ± 4.9	34.2 ± 5.5
Triglyceride (mmol/L)	1.56 ± 0.26	3.68 ± 0.24	1.86 ± 0.34
HOMA-IR	1.8 ± 0.4	13.4 ± 1.9	4.5 ± 1.4

Data indicated that mean ± SEM

#### The effect of endurance exercise with diet on liver weight and hepatic steatosis

**Fig. 4:**
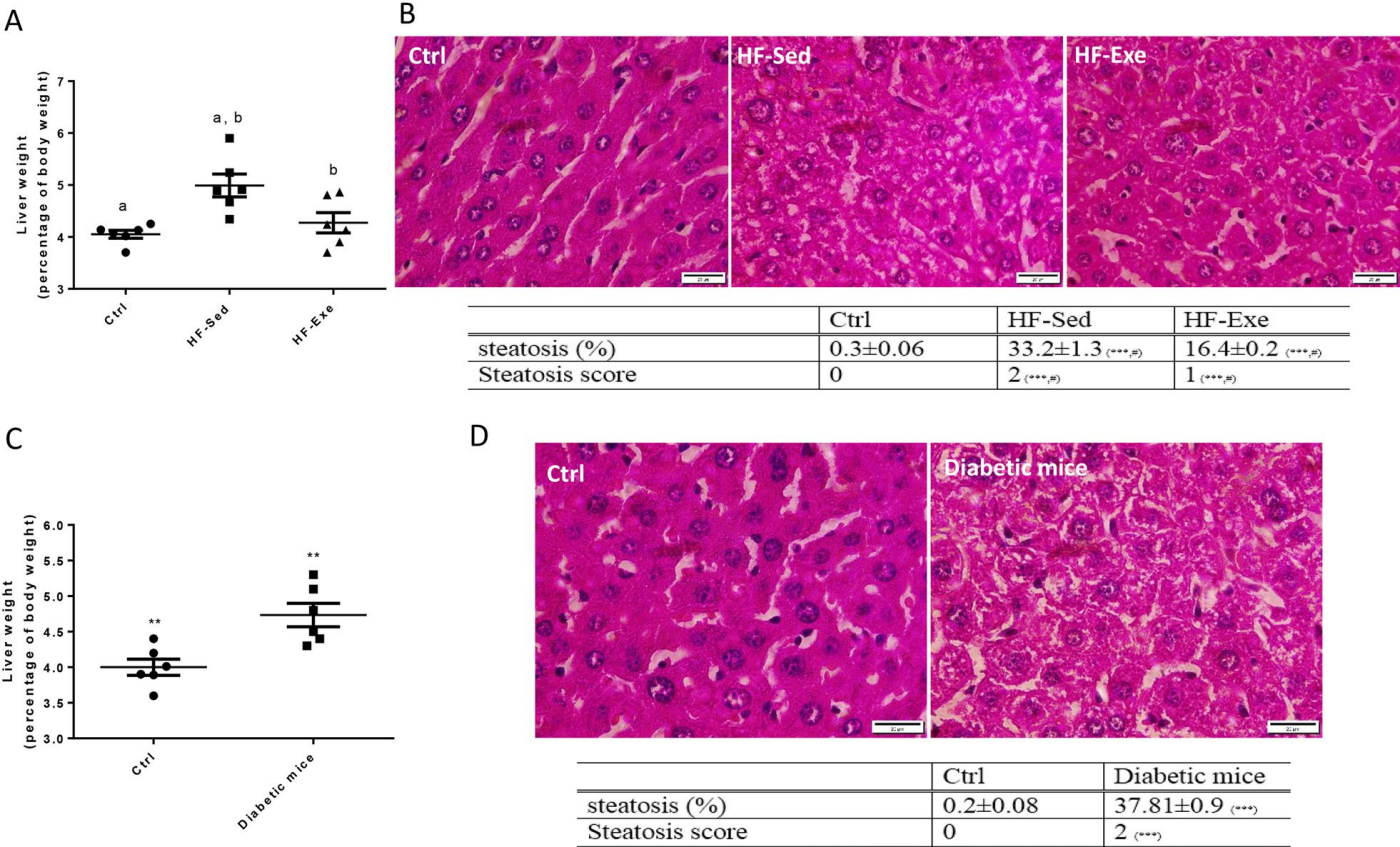
Effects of HF diet and exercise on liver weight and hepatic steatosis in all of the groups. **A** Liver weight (percentage of body weight) and **B** liver histology by H&E staining (scale bar: 20 μm) and steatosis score in prediabetic model mice. **C** Liver weight and **D** liver histology in diabetic model mice. ***p < 0.001 for HF-Sed and HF-Exe vs. Ctrl, ^#^*p* < 0.001 for HF-Exe vs. HF-Sed (**B**). ***p < 0.001 for diabetic mice vs. Ctrl, **p < 0.01 for diabetic mice vs. Ctrl (**D**). (a), and (b) represent a significant difference between (Ctrl and HF-Sed) and (HF-Exe and HF-Sed), respectively. All data are shown as means ± SEM, n = 6 per group

Mice on the HF diet developed hepatomegaly by week 12 high-fat diet (liver weight: HF-Sed (n = 6): 1.38 ± 0.05 g vs. Ctrl (n = 6): 1.08 ± 0.03 g, *p* < 0.01). Also, liver weight was decreased in HF-Exe group (n = 6): 1.19 ± 0.02 vs. HF-Sed ones (*p* < 0.01, Fig. 4A).

Histological evaluation of H&E-stained liver sections showed macrovesicular steatosis with few ballooned hepatocytes and minimal lobular inflammation in the HF-Sed mice, while there was a significantly lower degree of hepatic steatosis in the HF-Exe group (HF-Exe (n = 6): 16.4 ± 0.22% fat vs. HF-Sed (n = 6): 32.8 ± 1.4% fat, *p* < 0.05, Fig. 4B). While the steatosis score decreased significantly (HF-Exe (n = 6): 2.1 ± 0.2 vs. HF-Sed (n = 6): 3.2 ± 0.2, *p* < 0.05, Fig. 4B). These data suggest that the 8-week endurance exercise was an effective intervention to improve the histological hallmarks, predominantly hepatic steatosis of NAFLD.

Also, diabetic model mice developed hepatomegaly by week 12 HF diet and injection with STZ solution (liver weight: diabetic mice (n = 6): 1.25 ± 0.06 g vs. Ctrl (n = 6): 1.04 ± 0.05 g, (*p* < 0.01, Fig. 4C). Histological evaluation of liver sections showed macrovesicular steatosis with few ballooned hepatocytes and minimal lobular inflammation in diabetic model mice. (Diabetic mice (n = 6): 27.91 ± 0.39 vs. Ctrl (n = 6): 4.96 ± 0.08 fat, *p* < 0.001, Fig. 4D).

#### The expression levels of *Srebf1* mRNA and *GM38501* in prediabetic and diabetic model

**Fig. 5:**
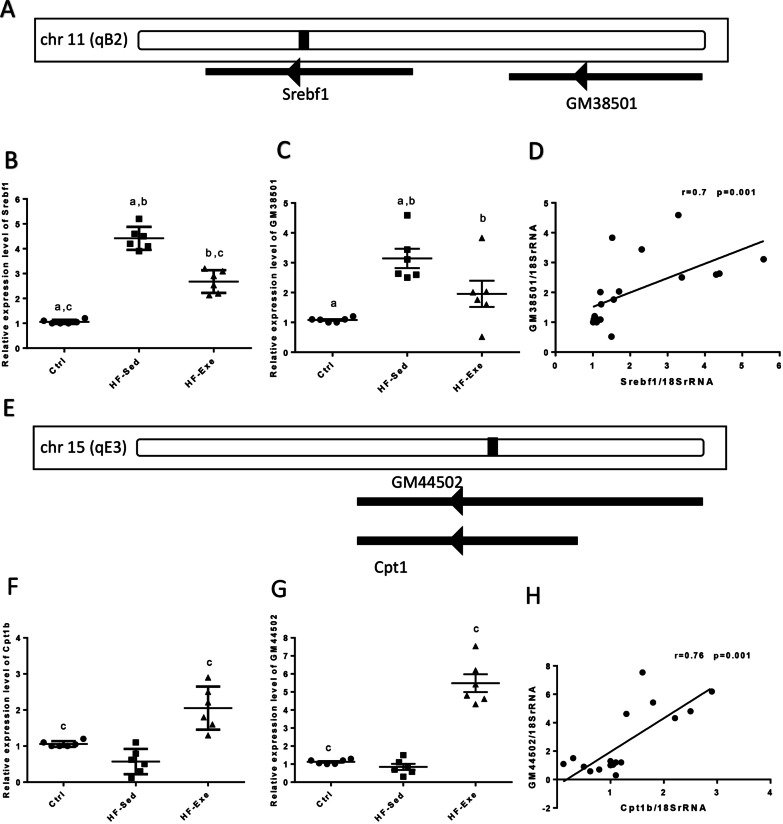

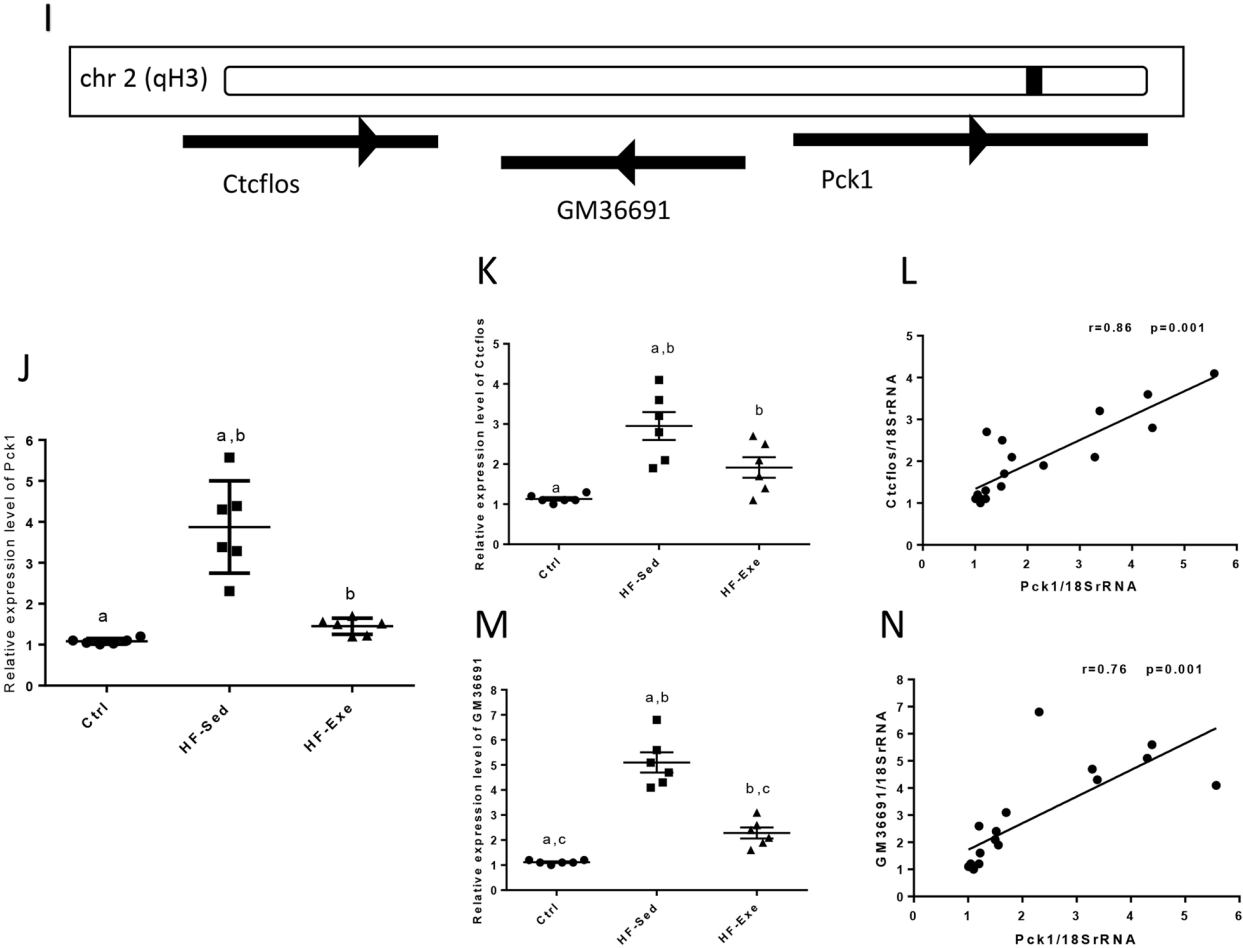
The expression levels of HML with transcript level of *18SrRNA* in prediabetic model mice. **A** Schematic chromosomal location, **B**, **C** Relative expression, and **D** correlation between expression levels of *Srebf1* and *GM38501*. **E** Schematic chromosomal location, (**F**, **G**) relative expression and **H** correlation between expression levels of *Cpt1b* and *GM44502*. **I** Schematic chromosomal location, **J**, **K** relative expression and **L** correlation between expression levels of *Pck1* and *Ctcflos*. **M** Relative expression levels of *GM36691* and **N** correlation between expression levels of *Pck1* and *GM36691*. All data are shown as means ± SEM, n = 6 per group. (a), (b), and (c) represent a significant difference between (Ctrl and HF-Sed), (HF-Exe and HF-Sed), and (Ctrl and HF-Exe), respectively. Data calculated by one-way ANOVA. Ctrl, Control; HF-Exe, high-fat diet-exercise; HF-Sed, high-fat diet-sedentary; *Srebf1, Sterol Regulatory Element Binding Transcription Factor 1*, *Cpt1b*, *Carnitine Palmitoyltransferase 1B*, *Pck1, Phosphoenolpyruvate Carboxykinase 1*

Chromosomal location of *Srebf1* and its predicted lncRNA *GM38501* is schematically illustrated (Fig. 5A). Hepatic *Srebf1* expression was increased in the prediabetic mice (HF-Sed) vs. their control group (*p* < 0.001) and was decreased in HF-Exe (Fig. 5B, p < 0.001). Also, the expression level of *GM38501* was significantly higher in the HF-Sed mice than the LF-fed ones (*p* < 0.01). It was significantly lower in the HF-Exe mice vs. prediabetic mice (Fig. 5C, p < 0.05). There was a significant positive correlation between *Srebf1* mRNA and *GM38501* expression in the prediabetic mice (Fig. 5D, r = 0.7, *p* = 0.001).

**Fig. 6:**
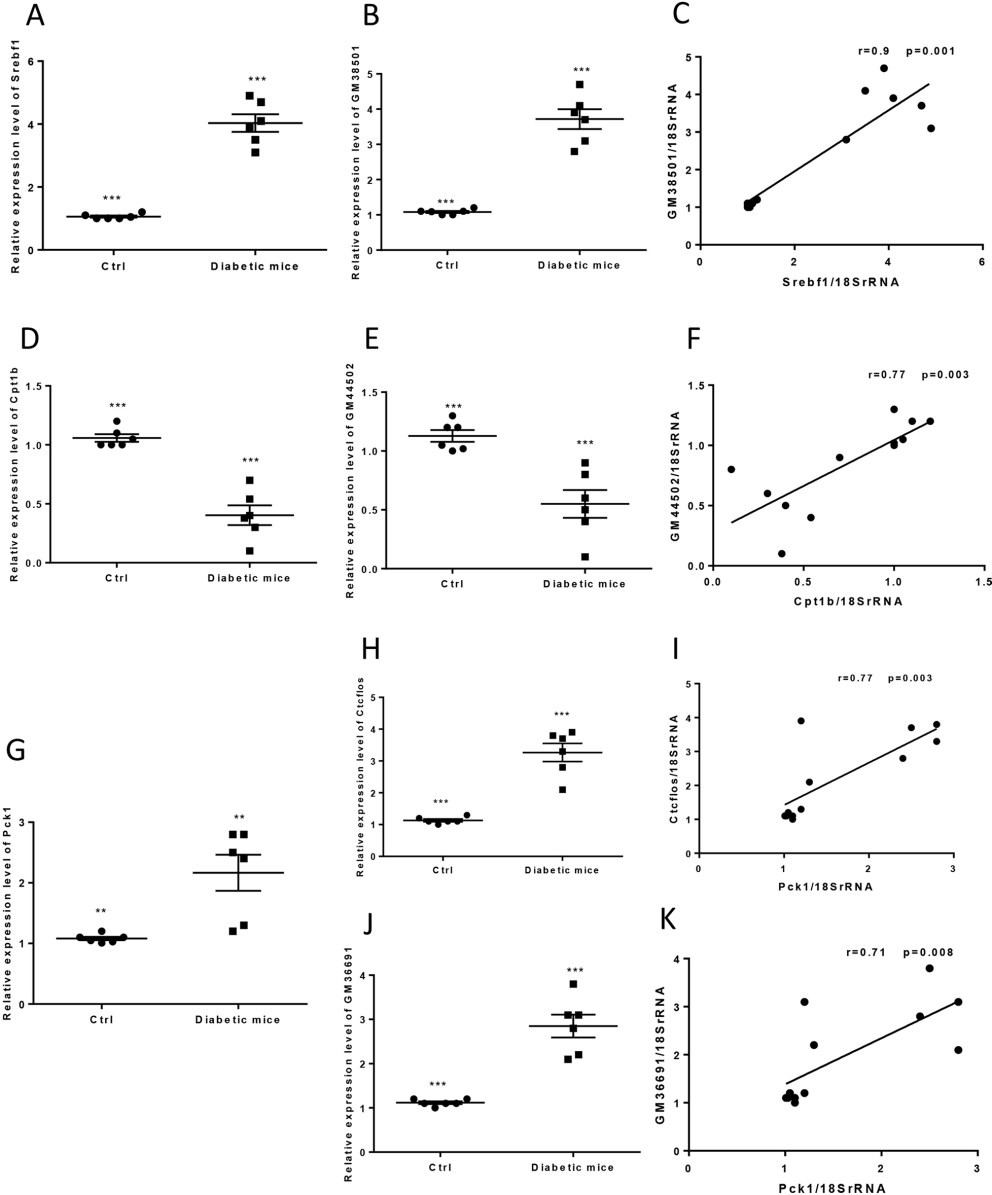
The expression levels of HML with transcript level of *18SrRNA* in diabetic model mice. **A**, **B** Relative expression and **C** correlation between expression levels of *Srebf1* and *GM38501*. **D**, **E** Relative expression and **F** correlation between expression levels of *Cpt1b* and *GM44502*. **G**, **H** Relative expression and **I** correlation between expression levels of *Pck1* and *Ctcflos*. **J** Relative expression levels of *GM36691* and **K** correlation between expression levels of *Pck1* and *GM36691*. All data are shown as means ± SEM, n = 6 per group. ***p < 0.001 for diabetic mice vs. Ctrl. Data calculated by one-way ANOVA. Ctrl, Control; HF-Exe, high-fat diet-exercise; HF-Sed, high-fat diet-sedentary

Also, expression levels of *Srebf1* and *GM38501* were significantly higher in the diabetic model mice than control group (Fig. 6A, B, p < 0.001). There was a significant positive correlation between *Srebf1* mRNA and *GM38501* expression in diabetic mice (Fig. 6C, r = 0.9, *p* = 0.001).

#### The expression levels of *Cpt1b* mRNA and *GM44502* in prediabetic and diabetic model

Chromosomal location of *Cpt1b* and its predicted lncRNA *GM44502* is schematically illustrated (Fig. 5E). Hepatic *Cpt1b* expression was decreased in the HF-Sed mice versus their control group, but there was not significant (*p* = 0.1). The relative expression level of *Cpt1b* was increased in the HF-Exe animals vs. prediabetic mice (*p* < 0.001) and vs. the LF-fed mice (Fig. 5F, p < 0.01). Also, the expression level of *GM44502* was lower in the HF-Sed mice in respect to the LF-fed ones, but there was not significant (*p* = 0.7). Hepatic *GM44502* expression was significantly higher in the HF-Exe mice vs. prediabetic mice (*p* < 0.001) and vs. control group (Fig. 5G, p < 0.001). There was a significant positive correlation between *Cpt1b* mRNA and *GM44502* expression in the prediabetic mice (Fig. 5H, r = 0.76, *p* = 0.001).

Also, the expression levels of *Cpt1b* and *GM44502* was significantly lower in the diabetic model mice vs. control group (Fig. 6D, E, p < 0.001). There was a significant positive correlation between *Cpt1b* mRNA and *GM44502* expression in the diabetic mice (Fig. 6F, r = 0.77, *p* = 0.003).

#### The expression levels of *Pck1* mRNA, *Ctcflos*, and *GM36691* in prediabetic and diabetic model

Chromosomal location of *Pck1* and its predicted lncRNA *Ctcflos* and *GM36691* is schematically illustrated (Fig. 5I). Hepatic *Pck1* expression was increased in the prediabetic mice (HF-Sed) than their control group (*p* < 0.001) and was decreased in HF-Exe vs. HF-Sed animal (Fig. 5J, p < 0.001). Also, expression level of *Ctcflos* was higher in the HF-Sed mice vs. the LF-fed ones *(p* < 0.001) and was significantly lower in the HF-Exe mice compare to prediabetic mice (Fig. 5K, p < 0.05). There was a significant positive correlation between *Pck1* mRNA and *Ctcflos* expression in prediabetic mice (Fig. 5L, r = 0.86, *p* = 0.001).

Also, expression level of *GM36691* was higher in the prediabetic mice versus the Ctrl group *(p* < 0.001) and was significantly lower in the HF-Exe mice vs. prediabetic mice (Fig. 5M, p < 0.05). There was a significant positive correlation between *Pck1* mRNA and *GM36691* expression in the prediabetic mice (Fig. 5N, r = 0.76, *p* = 0.001).

Also, expression levels of *Pck1, Ctcflos*, and *GM36691* were significantly higher in the diabetic model mice vs. control group (Fig. 6G, H, J, p < 0.01 for *Pck1* and *p* < 0.001 for *Ctcflos* and *GM36691*). There was a significant positive correlation between *Pck1* mRNA and *Ctcflos* expression in the diabetic mice (Fig. 6I, r = 0.77, *p* = 0.003), and there was a significant positive correlation between *Pck1* mRNA and *GM36691* expression in the diabetic mice (Fig. 6K, r = 0.71, *p* = 0.008).

## Discussion

This study was partly done by bioinformatics tools regarding to the screening of hepatic lncRNAs juxtaposition to genes involved in prediabetes key pathways. We predicted association of hepatic mRNAs -lncRNAs (HML) of genes. Accordingly, if an lncRNA was located in 50 kb proximity of an mRNA, its expression was considered to be correlated with the respective gene and a potential regulatory role was assumed between them [23, 24]. In order to candidate the input lncRNAs set and mRNAs set for the screening, we selected two available high throughput microarray datasets resulted from studies that had fed mice similarly to our feeding design with a long term high-fat diet to induce pathophysiological metabolic conditions. Besides, we gathered literature- and database-extracted genes and lncRNAs associated with prediabetes conditions to have an inclusive repository of input data. Using Python programming, we checked the matching of the differentially expressed genes and lncRNAs in the aforementioned datasets in terms of their chromosomal location with a threshold of 50 kb in juxtaposition. We narrowed down the results in terms of the importance of metabolic pathways which were enriched for the genes in prediabetes and annotation of the lncRNAs in genome browser databases. Finally, we shortlisted three predicted HML pairs and examined their expression in prediabetic mice induced with a long term HF diet. Results showed significant upregulation of *Srebf1* associated with *GM38501*, *Pck1* associated with *Ctcflos*, and *GM36691*, and a positive correlation was observed between all predicted HML pairs.

In recent studies, researchers have reported that lncRNAs are associated with the prevalence of diabetes and insulin resistance is one of the most important indicators in prediabetes. Carter and colleagues (2015) demonstrated that lncRNA GAS5 as a significant indicator of diabetes which can be easily measured in serum in a cohort of US military veterans. This study showed that GAS5 levels may predict onset of diabetes in adults [39]. Also, Zhang et.al (2018) concluded that lncRNA MEG3 overexpression may inhibit the development of diabetic retinopathy by inhibiting TGF-β1 and VEGF expression [40]. In another study, researchers reported that exercise reduced insulin resistance in type 2 diabetes mellitus via mediating the lncRNA MALAT1/microRNA-382-3p/resistin axis [41]. The results of a study showed that lncRNA *Ctcflos* affects metabolism by regulating transcription [42].

Obesity and diabetes are among the key risk factors of NAFLD [43, 44]. In this study, bioinformatics clustering annotation analysis showed that NAFLD is strongly enriched in gene networks associated with HF-induced pathophysiological metabolic conditions. Our histological results confirmed the incidence of NAFLD in prediabetic mice, as well as amelioration of steatosis score in the exercised group. Our data showed that expression patterns of the regulatory network have significant discrimination power for steatosis score through the groups of Ctrl, Prediabetes, Diabetes, and Exercised mice (Additional file 5: Table 2).

**Fig. 7:**
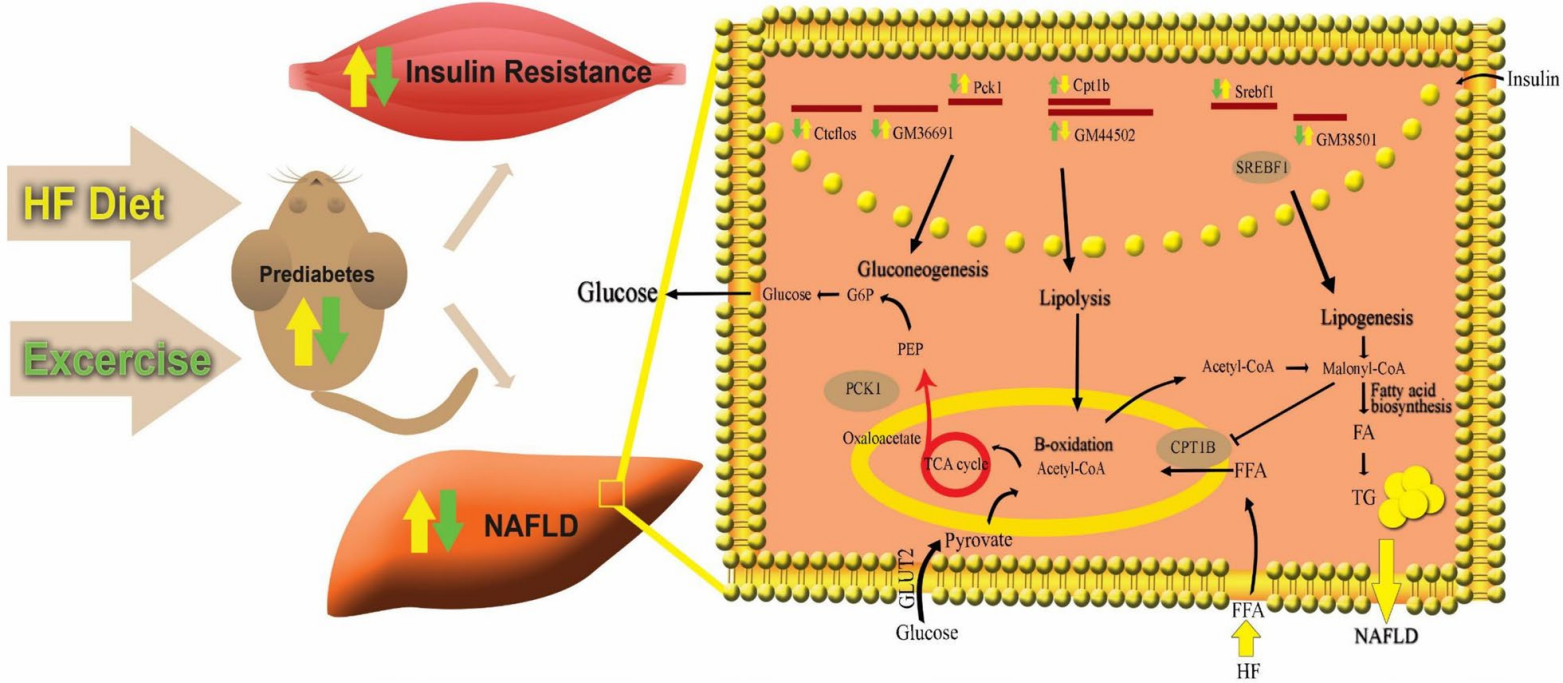
In this study, HF diet-induced prediabetes, insulin resistance, and NAFLD downregulating *Cpt1b* and upregulating *Srebf1*, *Pck1*, and positively correlated to their predicted lncRNAs included *GM44502*, *GM38501*, *Ctcflos*, *GM36691*, respectively. These three genes are key players in Lipogenesis, Lipolysis, and Gluconeogenesis pathways, respectively. These pathways have been altered in NAFLD and insulin resistance

The liver plays a key role in adaptation to glucose levels and affects energy homeostasis of other tissues, including muscles by modulating glucose levels [45, 46]. On the other hand, insulin stimulates synthesis of cholesterol and triglyceride in liver as well as glucose uptake into peripheral tissues such as muscle and fat. Notably, insulin-resistant adipocyte contributes the further hepatic triglyceride synthesis. Thus, the paradox in hepatic insulin resistance is that insulin fails to suppress hepatic glucose production, nevertheless it continues to stimulate lipogenesis, causing hyperglycemia, hyperlipidemia, hepatic steatosis, and T2DM [47]. In the HF-liver (HF-diet fed mice liver), the HML network, including *Ctcflos*, *GM36691* associated with *Pck1*, is upregulated, representing that gluconeogenesis pathway is increased, potentially leading to an increase of glucose level in blood. In these conditions, glucose consuming tissues such as skeletal muscle, which are affected by the obesity-induced insulin resistance, could not be able to uptake the blood glucose [6]. Simultaneously, the HF-liver HML network, including *GM38501* associated with *Srebf1* is elevated, demonstrating that lipogenesis occurs producing triglyceride in the hepatocytes. Concurrently, the HF-liver HML network, including *GM44502* associated with *Cpt1b*, is downregulated, indicating that the lipolysis pathway is decreased. As well studies have made evident that, the Malonyl-CoA produced in the lipogenesis pathway, suppresses the activity of CPT1B, inhibiting the transportation of fatty acids to the mitochondria by CPT1B, resulting in an accumulation of TGs and FAs in the hepatocytes and the subsequent development of NAFLD (Fig. 7).

The remarkable thing is that the study Bonilauri (2020) reported that exercise-induced lncRNAs correlated with genes in these pathways: collagen fibril organization, ECM organization, protein complex subunit organization, synaptic transmission (cholinergic), long-term synaptic potentiation, regulation of CDK activity, skeletal system development, and plasma and myoblast fusion [48]. In Jafarinejad Farsang's study (2021) was shown that the beneficial effect of Exercise on the improvement of cardiac function and reduction of fibrosis in infarcted heart possibly through regulation of the expression of lncRNAs: H19, GAS5, and MIAT [49].

Interestingly, when the prediabetic mice were trained with an aerobic exercise and decreased the body mass, the patterns of expressions in the HML network were reversed, signifying the potential of this regulatory network to be targeted in future precision-based therapeutic approaches. Also, in response to exercise, ATP turnover is increased, resulting in increased ATP consumption and a rise in intracellular AMP levels. These changes lead to the activation of AMPK in rodents and in human muscle. AMPK regulates multiple signaling pathways whose overall effects are to increase ATP production, including fatty acid oxidation and glucose uptake. We suggest that activation of the AMPK pathway and the cell needs for energy may lead to changes in the hepatic mRNAs-lncRNAs (HML) network.

## Conclusions

We suggest that the expression patterns of the HML network involved in IR and NAFLD was significantly modified in prediabetes. These phenomena may trigger a cascade of further complications, if ignored, promote diabetic condition. Thus, here we proposed IR and NAFLD networks as prerequisite conditions in prediabetes which potentially denote pathogenic pathways in diabetes. Therefore, special care should be dedicated to these signaling pathways in regard to preventing diabetic state.

## Supplementary Information

**Additional file 1: Fig. 1.** Bioinformatics pipeline of the study showing entries sets including lncRNAs and mRNAs screened by differential analysis of GEO datasets (GSE85439, GSE94790), MGD database, and literature review. Coding genes were screened by their involvement in pathways associated with prediabetes. Python programming was used to obtain mRNAs-lncRNAs pair locating in 50 kb proximity (HML). The resulted set was analyzed and enriched in biological pathways related to lipids and carbohydrates metabolism. Final selected pairs were subjected to experimental evaluation of the expression. DEA: Differential expression analysis. MGD: Mouse genome database.

**Additional file 2: Fig. 2.** Insulin resistance pathway in the liver. http://www.kegg.jp/kegg/kegg1.html.

**Additional file 3: Fig. 3.** Body weight, blood glucose, and insulin monitored throughout the treatment of HF diet and stz injection. (A) Changes in body weight in control and diabetic mice. (B) Glucose tolerance test (GTT) on control and diabetic mice after 12 weeks intervention. (C) Blood glucose AUC values for GTT. (D) Insulin tolerance test (ITT) on control and diabetic mice after 12 weeks intervention. (E) Blood glucose AUC values for ITT. *p < 0.05. All data are shown as means ± SEM, n = 6 per group.

**Additional file 4: Table 1.** List of the mRNA and lncRNA primer sequences.

**Additional file 5: Table 2.** Receiver operating characteristic (ROC) data of variables displaying discrimination power for one of top lncRNAs (Gm38501) in calculation of NAFLD activity score (NAS) given as area under the curve (AUC) and 95% confidence interval (CI).

## Data Availability

The data set used and analyzed during the current study are available for the corresponding authors on a reasonable request.

## Abbreviations

IR: Insulin resistance
LncRNA: Long non-coding RNA
HML: Hepatic mRNAs-lncRNAs
STZ: Streptozocin
GTT: Glucose tolerance test
LF: Low fat
Ctrl: Control
HbA1c: Glycated hemoglobin
HF: High-fat
HF-Exe: High fat diet-Exercised
HF-Sed: High fat diet-Sedentary
RT-qPCR: Reverse transcription-quantitative polymerase chain reaction
PCR: Polymerase chain reaction
SEM: The standard error of the mean
*Srebf1*: *Sterol Regulatory Element Binding Transcription Factor 1*
*Cpt1b*: *Carnitine palmitoyltransferase 1B*
*Pck1*: *Phosphoenolpyruvate Carboxykinase 1*
*18SrRNA*: *18S ribosomal RNA*
MGD: Mouse genome database
