# Supplementary figures and images for: Modulation and bioinformatics screening of hepatic mRNA-lncRNAs (HML) network associated with insulin resistance in prediabetic and exercised mice
Source: Nutr Metab (Lond). 2021 Jul 20;18:75. doi: 10.1186/s12986-021-00600-0 (PMC8290563; doi:10.1186/s12986-021-00600-0)

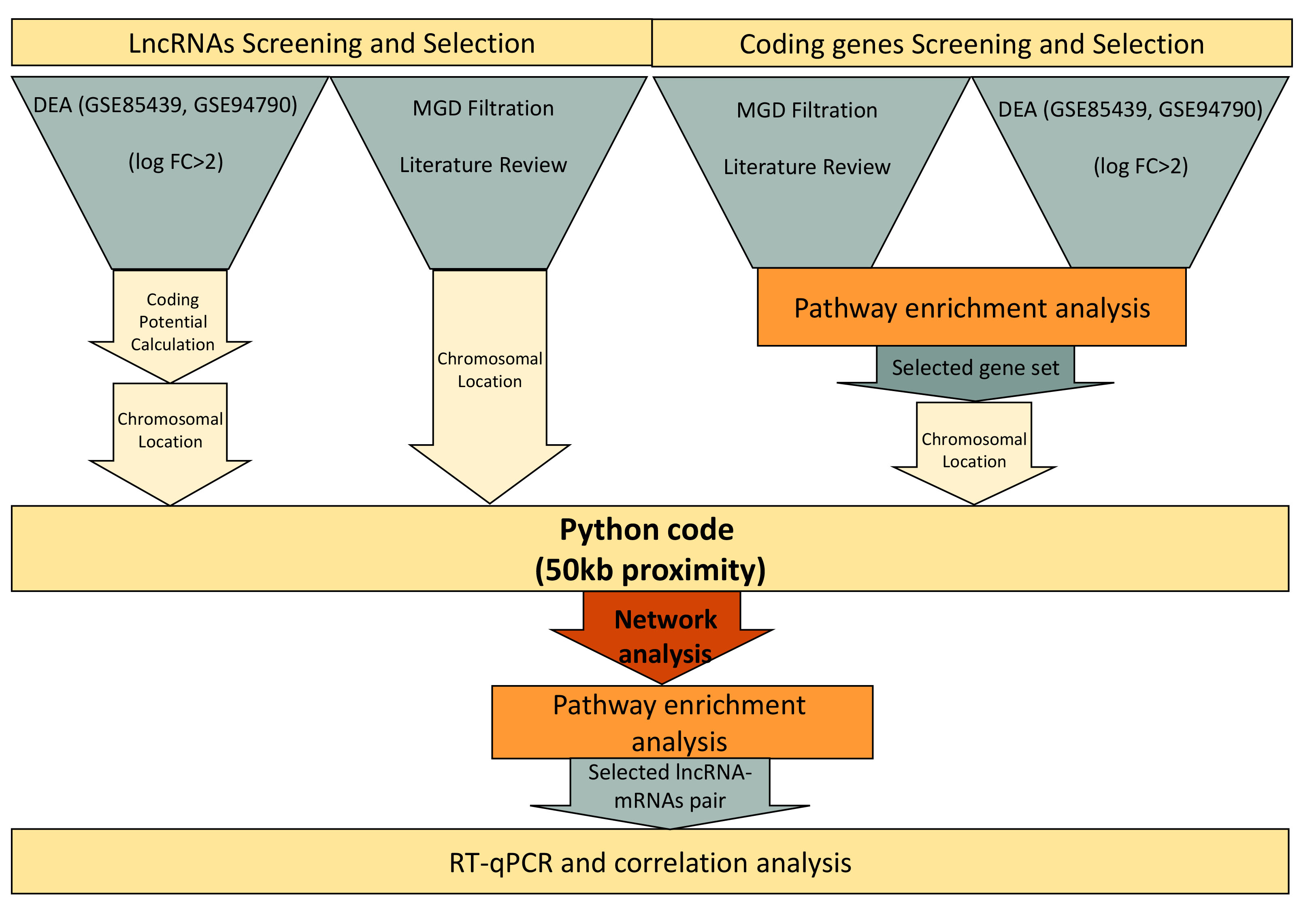

Supplement: Supplementary file 1 — Additional file 1: Fig. 1. Bioinformatics pipeline of the study showing entries sets including lncRNAs and mRNAs screened by differential analysis of GEO datasets (GSE85439, GSE94790), MGD database, and literature review. Coding genes were screened by their involvement in pathways associated with prediabetes. Python programming was used to obtain mRNAs-lncRNAs pair locating in 50 kb proximity (HML). The resulted set was analyzed and enriched in biological pathways related to lipids and carbohydrates metabolism. Final selected pairs were subjected to experimental evaluation of the expression. DEA: Differential expression analysis. MGD: Mouse genome database. [file 12986_2021_600_MOESM1_ESM.jpg]

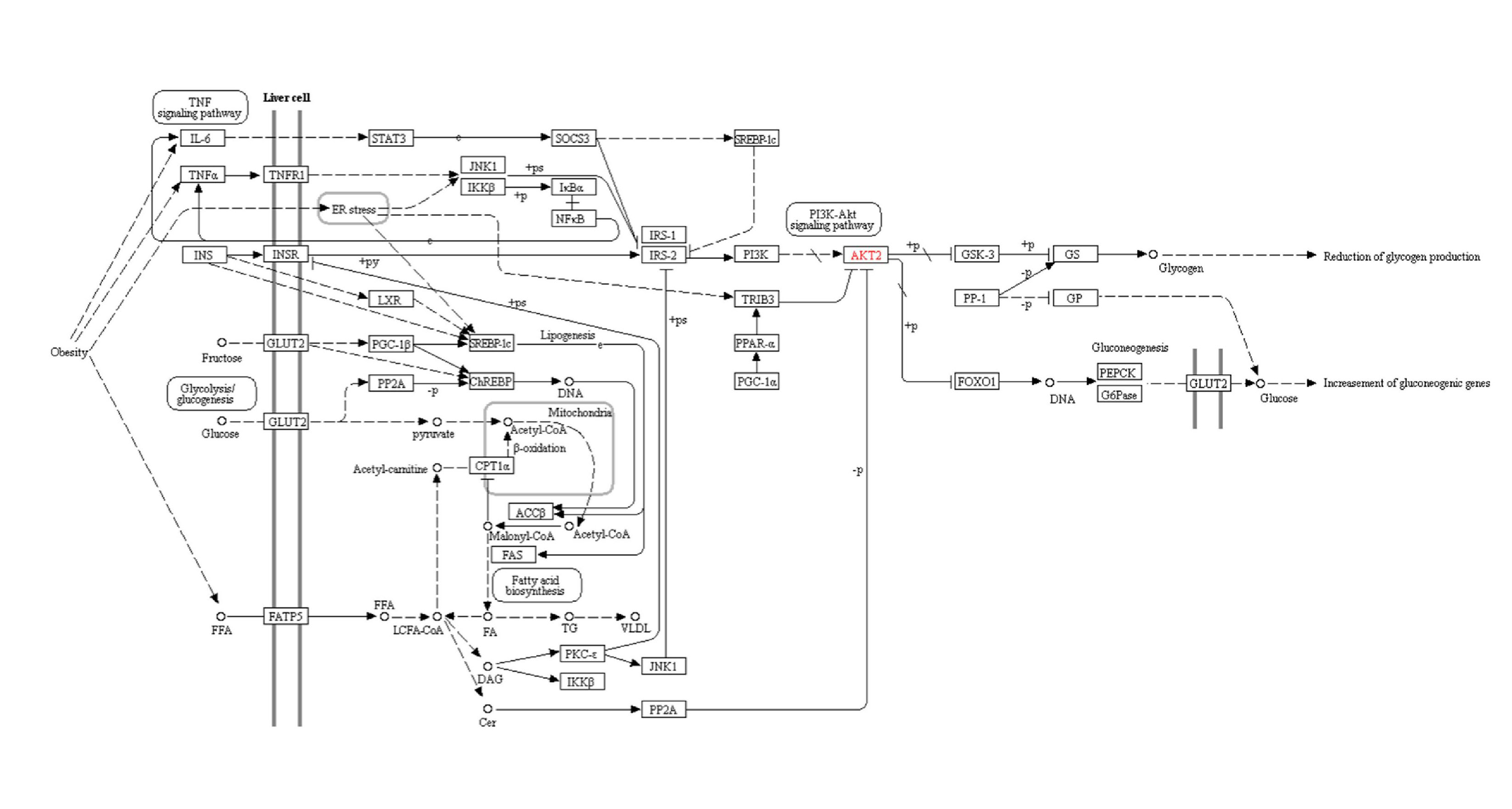

Supplement: Supplementary file 2 — Additional file 2: Fig. 2. Insulin resistance pathway in the liver. http://www.kegg.jp/kegg/kegg1.html. [file 12986_2021_600_MOESM2_ESM.jpg]

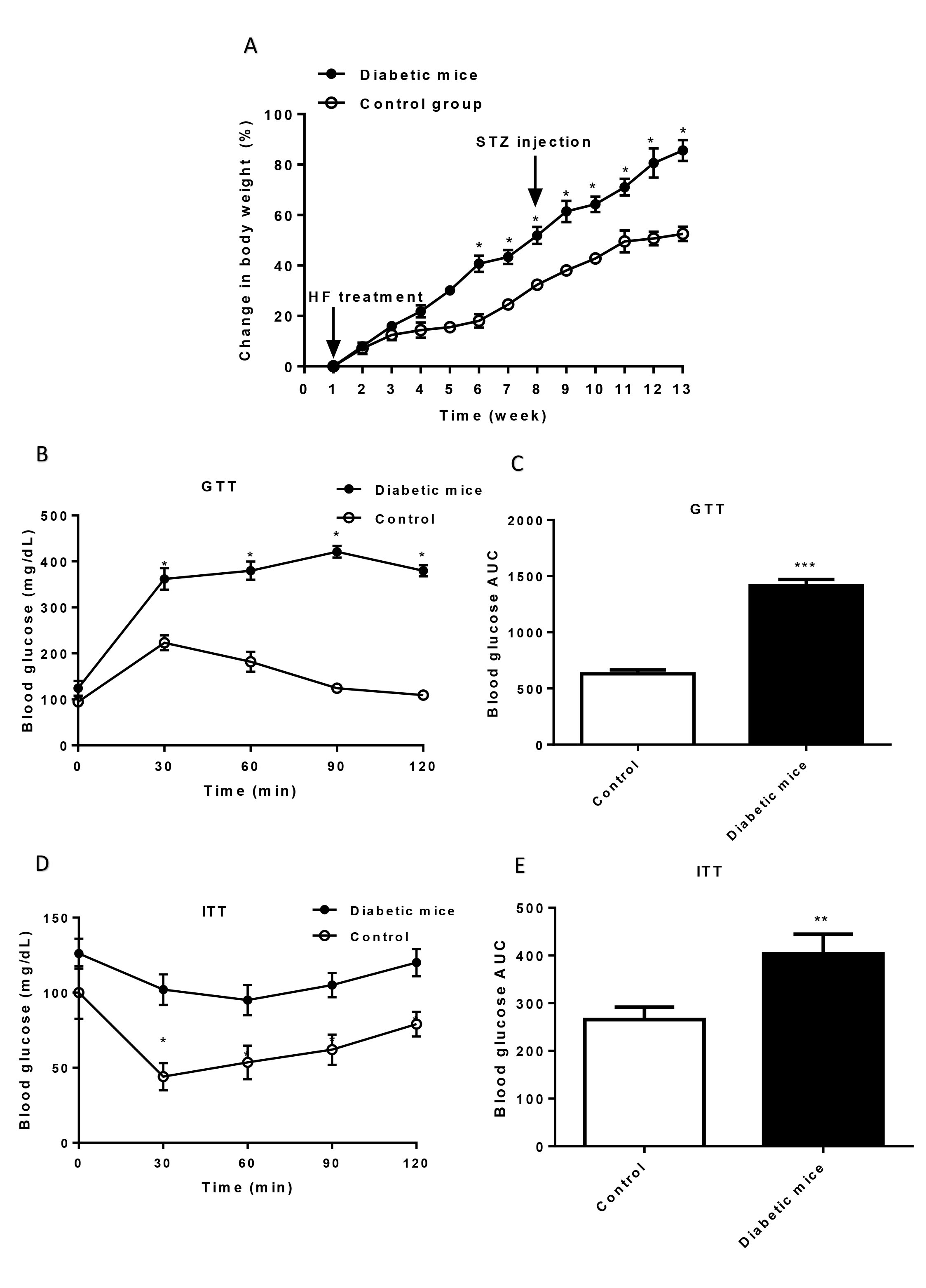

Supplement: Supplementary file 3 — Additional file 3: Fig. 3. Body weight, blood glucose, and insulin monitored throughout the treatment of HF diet and stz injection. (A) Changes in body weight in control and diabetic mice. (B) Glucose tolerance test (GTT) on control and diabetic mice after 12 weeks intervention. (C) Blood glucose AUC values for GTT. (D) Insulin tolerance test (ITT) on control and diabetic mice after 12 weeks intervention. (E) Blood glucose AUC values for ITT. *p < 0.05. All data are shown as means ± SEM, n = 6 per group. [file 12986_2021_600_MOESM3_ESM.jpg]
