# Supplementary material for: Modulation and bioinformatics screening of hepatic mRNA-lncRNAs (HML) network associated with insulin resistance in prediabetic and exercised mice
Source: Nutr Metab (Lond). 2021 Jul 20;18:75. doi: 10.1186/s12986-021-00600-0 (PMC8290563; doi:10.1186/s12986-021-00600-0)
Supplement: Supplementary file 4 — Additional file 4: Table 1. List of the mRNA and lncRNA primer sequences. [file 12986_2021_600_MOESM4_ESM.docx]

**Supplemental Table 1.** List of the mRNA and lncRNA primer sequences

| Genes | Forward primer (5´–3´) | Reverse primer (5´–3´) | | | Product size (bp) |
| --- | --- | --- | --- | --- | --- |
| *Srebf1* | CACTTCTGGAGACATCGCAAAC | ATGGTAGACAACAGCCGCATC | | | 282 |
| *Cpt1b* | AAACTGGACCGTGAAGAGAT | TTGCCTGGGATGCGTGTA | | | 104 |
| *Pck1* | AAGCATTCAACGCCAGGTTC | GGGCGAGTCTGTCAGTTCAAT | | | 122 |
| *GM38501* | TCAGAGTAGAAGAGGCAGAAAC | | | GCAGAAGAAGGGCGACATA | 90 |
| *GM44502* | GCCTCAAAGTGCTGAATAAACC | AATAAGGCGTTTCTTCCAGG | | | 159 |
| *Ctcflos* | CATCGGTGGAATCAAGAAGTT | CAGGCAGTGTCATAAAGTGTT | | | 135 |
| *GM36691* | TAGAGCAGTTAGGAAGCAAGA | ATGTATTATGTAGCCAAGGATGAC | | | 166 |
| *18SrRNA* | CGGACACGGACAGGATTG | | TCGCTCCACCAACTAAGAAC | | 85 |
