# Supplementary material for: Modulation and bioinformatics screening of hepatic mRNA-lncRNAs (HML) network associated with insulin resistance in prediabetic and exercised mice
Source: Nutr Metab (Lond). 2021 Jul 20;18:75. doi: 10.1186/s12986-021-00600-0 (PMC8290563; doi:10.1186/s12986-021-00600-0)
Supplement: Supplementary file 5 — Additional file 5: Table 2. Receiver operating characteristic (ROC) data of variables displaying discrimination power for one of top lncRNAs (Gm38501) in calculation of NAFLD activity score (NAS) given as area under the curve (AUC) and 95% confidence interval (CI). [file 12986_2021_600_MOESM5_ESM.docx]

**Supplemental Table 2.** Receiver operating characteristic (ROC) data of variables displaying discrimination power for one of top lncRNAs (Gm38501) in calculation of NAFLD activity score (NAS) given as area under the curve (AUC) and 95% confidence interval (CI).

| **HF-Sed *vs*. HF-Exercise** | **Ctrl *vs*. HF-Sed** | **Ctrl *vs*. Diabetes** | **Gm38501** |
| --- | --- | --- | --- |
| 2 *vs*. 1 | 0 *vs*. 2 | 0 *vs*. 2 | **Steatosis score** |
| < 2.265 | *> 1.850* | *> 2.000* | **Cutoff point** |
| 0.0374 | 0.0039 | 0.0039 | **P-value** |
| 0.8611 **±**  0.6036 to 1.119 | 1.000 **±**  1.000 to 1.000 | 1.000 **±**  1.000 to 1.000 | **Area ± 95%CI** |
| 83.33 **±**  35.88% to 99.58% | 100.0 **±**  54.07% to 100.0% | 100.0 **±**  54.07% to 100.0% | **Sensitivity ± 95%CI** |
| 100.0 **±**  54.07% to 100.0% | 100.0 **±**  54.07% to 100.0% | 100.0 **±**  54.07% to 100.0% | **Specificity ± 95%CI** |

| **HF-Sed vs. HF-Exercise** | **Ctrl vs. HF-Sed** | **Ctrl vs. Diabetes** | **Gm36691** |
| --- | --- | --- | --- |
| 2 vs. 1 | 0 vs. 2 | 0 vs. 2 | **Steatosis score** |
| < 3.600 | > 2.650 | > 1.650 | **Cutoff point** |
| 0.0039 | 0.0039 | 0.0039 | **P-value** |
| 1.000 **±**  1.000 to 1.000 | 1.000 **±**  1.000 to 1.000 | 1.000 **±**  1.000 to 1.000 | **Area ± 95%CI** |
| 100.0 **±**  54.07% to 100.0% | 100.0 **±**  54.07% to 100.0% | 100.0 **±**  54.07% to 100.0% | **Sensitivity ± 95%CI** |
| 100.0 **±**  54.07% to 100.0% | 100.0 **±**  54.07% to 100.0% | 100.0 **±**  54.07% to 100.0% | **Specificity ± 95%CI** |

| **HF-Sed vs. HF-Exercise** | **Ctrl vs. HF-Sed** | **Ctrl vs. Diabetes** | **Ctcflos** |
| --- | --- | --- | --- |
| 2 vs. 1 | 0 vs. 2 | 0 vs. 2 | **Steatosis score** |
| < 2.750 | > 1.600 | > 1.700 | **Cutoff point** |
| 0.0453 | 0.0039 | 0.0039 | **P-value** |
| 0.8472 **±**  0.6185 to 1.076 | 1.000 **±**  1.000 to 1.000 | 1.000 **±**  1.000 to 1.000 | **Area ± 95%CI** |
| 100.0 **±**  54.07% to 100.0% | 100.0 **±**  54.07% to 100.0% | 100.0 **±**  54.07% to 100.0% | **Sensitivity ± 95%CI** |
| 66.67 **±**  22.28% to 95.67% | 100.0 **±**  54.07% to 100.0% | 100.0 **±**  54.07% to 100.0% | **Specificity ± 95%CI** |

| **HF-Sed vs. HF-Exercise** | **Ctrl vs. HF-Sed** | **Ctrl vs. Diabetes** | **Gm44502** |
| --- | --- | --- | --- |
| 2 vs. 1 | 0 vs. 2 | 0 vs. 2 | **Steatosis score** |
| > 2.910 | < 1.010 | < 0.9500 | **Cutoff point** |
| 0.0039 | 0.1495 | 0.0039 | **P-value** |
| 1.000 **±**  1.000 to 1.000 | 0.7500 **±**  0.4313 to 1.069 | 1.000 **±**  1.000 to 1.000 | **Area ± 95%CI** |
| 100.0 **±**  54.07% to 100.0% | 66.67 **±**  22.28% to 95.67% | 100.0 **±**  54.07% to 100.0% | **Sensitivity ± 95%CI** |
| 100.0 **±**  54.07% to 100.0% | 83.33 **±**  35.88% to 99.58% | 100.0 **±**  54.07% to 100.0% | **Specificity ± 95%CI** |
